# Removal of Malignant Tumors of the Spinal Cord

**Published:** 1912-09

**Authors:** Kuwasaku Kato

**Affiliations:** (Japan); Inaugural Thesis, Georg-August University, Gottingen


### Removal of Malignant Tumors of the Spinal Cord

KUWASAKU KATO (Japan)
Inaugural Thesis, Georg-August University, Gottingen

The author gives in detail the clinical notes and result of an operation for removal of a malignant tumor of the spinal cord. A spindle celled sarcoma at the level of the 8th Dorsal vertebra was removed by Brann. Pressure symptoms disappeared and after a prolonged convalescence the patient remained well for 2 years. Then a recurrence of the tumor with pressure symptoms necessitated a second operation which was again followed by fair restoration of function. 3½ years after the second operation the patient died from recurrence of the tumor complicated by diabetes and pneumonia.

The author was able to find thirty other cases recorded of malignant tumors of the spinal cord. In none of these was permanent recovery reported. A brief abstract of the thirty cases is included in the paper.

---

Breast Affections: 100 cases, Randolph and Nathan Winslow, Baltimore, *Maryland Med. Jour.*, Dec., 1911. 62 were carcinoma; 3 sarcoma; 20 fibroadenoma; 3 adeno-cystoma; 1 adeno-fibro-cystoma; 1 cystic fibroma; 2 galactoceles; 3 tubercular mastitis; 1 circumcanilicular fibro-myxoma; 4 abscess. All but one were females, the man had a fibro-adenoma. 67 women were married, 27 single, 4 not stated. Right breast affected in 40, left in 58, 2 not stated. 94 operated upon, two deaths, one quite suddenly from unknown cause, the other from pneumonia. 2 refused operation and in 4 the case was too far advanced.

Of the cancer cases, three were under 35, 17, 26, and not stated. Seven more were under 40; the oldest was 82. The authors insist on the necessity of microscopic examination, citing errors in both directions.

